# An Avoided Danger to Voluntary Hospitals

**Published:** 1916-12-23

**Authors:** 


### AN AVOIDED DANGER TO VOLUNTARY HOSPITALS.

Knowledge is power, but experience since the war proves that what passes for knowledge in peace-time may penetrate very little, if at all, below the surface. One of the surprises of the war for those who have had most to do with hospital and institutional finance has been, the enormous outpouring of voluntary gifts for all sorts of objects and purposes connected with the war. Great as these have been, they are small matters compared with the enormous sums raised by taxation. All these things go to prove that the former idea that there was an average fixed sum which charity could yield each year, and that any attempt to increase it must fail, if it did not even deserve to fail, nowadays, is demonstrably nonsense. Those who possess large means are shy to admit the possession of wealth. After all, every person has wealth who can readily meet all the claims upon him and yet have in hand a surplus of revenue which steadily accumulates year by year. These points should be noted by the treasurer of every hospital, and remembered after the war.

Before the war it was always felt by the initiated that the field of charity offered no such promising ground, on which to base an appeal, as the needs of a voluntary hospital. The wayfaring man, who meets with an accident in the ordinary course of life's journey, has ever been one who appealed strongly to the sympathy and support of every well-disposed, benevolent person. It follows that hospital appeals, prepared with knowledge and acumen, have always yielded large and increasing returns. An examination of the figures of the voluntary hospitals in Great Britain for nineteen years, up to and including the year 1914, shows that, although the total annual expenditure has increased by £2,014,000, the income of these hospitals has increased, too, by £2,055,000, showing a surplus income of £41,000. Could anything better demonstrate the wonderful power which voluntary hospital appeals have in loosening the purse-strings of the benevolent? The whole of the years to which these figures apply were years of peace and plenty, except the last five war months of 1914, which had no serious effect upon the income of the hospitals. It thus appears that voluntary hospital finance rested upon a sound basis at the commencement of the present war.

The war, however, brought many claims for an ever-increasing number of hospital beds for the reception of warriors who were sick or wounded. This has resulted in something like a revolution in the methods of relief and the diminished number of beds left available for civil cases. The Government, after some hesitation, agreed to pay from 3s. to 4s. per head per diem for each warrior admitted to treatment in the voluntary hospitals. These sums have proved to be inadequate, but the admission of the wounded from the war has added still further to the popularity of the voluntary hospitals. Indeed, appeals on the ground that wounded were under treatment have proved so fruitful as to tempt the unwary to make this new feature the paramount claim put forward for public support. Fortunately, those responsible for the financial management of voluntary hospitals have shown wisdom in relying mainly on the old pleas, when seeking increased funds. To have done otherwise must have been to sow tares in the vineyard by weakening the force and diminishing the yield of voluntary hospital appeals after the war. Our readers, with these facts before them, will appreciate the novel methods pursued by the League of Mercy, as explained by the speakers at the annual meeting on the 19th instant (see page 253). Some of these appear not to be in harmony with the conservative and wise policy pursued by hospital treasurers generally throughout the nation.

